# Investigating the Role of English as a Foreign Language Learners’ Academic Motivation and Language Mindset in Their Grit: A Theoretical Review

**DOI:** 10.3389/fpsyg.2022.872014

**Published:** 2022-05-10

**Authors:** Yiran Liu

**Affiliations:** School of Foreign Languages, Henan Institute of Technology, Xinxiang, China

**Keywords:** English as a foreign language learners, academic motivation, grit, broaden-and-build theory, language mindset

## Abstract

This review made a critical attempt to examine the studies on the role of English as a foreign language (EFL) learners’ academic motivation and growth mindsets in their grit. Some investigations have been done on the role of academic motivation in learner grit. However, a significant positive correlation between academic motivation and grit has been approved in related studies. The related literature review justified the results by broaden-and-build and expectancy-value theories. The related literature has shown that grittier learners persist in doing tasks, and developing their intrinsic motivation. Furthermore, the related literature has approved the effect of learners’ language mindset on their grit. In other words, learners with a growth mindset are persistent, and they devote their time to their performance. Finally, the pedagogical implications are expanded to promote the quality of language learning. This review also provides some suggestions for further research to illuminate our perspectives over motivation, mindset, and their interactions with each other.

## Introduction

According to Weiner (1985), “In educational contexts, success is often attributed to high capability and hard work, and failure is credited to low capability and lack of trying” (p. 15). Learners with lower levels of language proficiency regularly ascribed their failure in language learning to internal causes (Elizondo, 2013). There have been some studies that examined the relationship between motivation, grit, and their effect on growth mindset (Saunders, 2013). Grit consists of two lower-order components, perseverance of effort and consistency of interests (Duckworth et al., 2007). The first sub-construct, perseverance of effort, indicates an ability to maintain effort for a long time in the face of difficulty or failure. The second sub-construct, consistency of interests, refers to the ability to sustain interests for a long time despite challenges or setbacks. The distinction between the two separate constructs is manifested in the Grit Scale and the Short Grit Scale (Duckworth et al., 2007). Grit is also being particularly emphasized by numerous educators, and it is regarded as one of the significant features that anyone should possess to be effective in their life (Tough, 2012). Grit has been studied in psychology (Sharkey et al., 2018; Vela et al., 2018; Fosnacht et al., 2019). Dörnyei (2001) stated that motivation is regarded as a critical emotional state that affects foreign language learning achievement. Many investigations have been done on the influence of various kinds of motivation on academic performance (Arabmofrad et al., 2019; Karabatak and Polat, 2020; Mammadov et al., 2021). Mindset, as another emotional state, is the belief of learners in dynamic or static states of intelligence in the process of learning (Plaks and Stecher, 2007). Learners’ emotional states in language learning contexts and their effect on language learning performance have drawn the attention of researchers. English as a foreign language learners’ emotional states such as motivation, mindset, and grit have played influential roles in foreign language learning. More studies are necessary for positive psychology concepts that concentrate on grit and other emotional states, particularly in challenging tasks in language learning. Moreover, language mindset and grit are of significant importance since they depend on learners’ level of perseverance, not on intellectual skills, which is manageable and variable. This study is significant since learners and teachers can be aware of emotional states in their academic performance.

## Review of Literature

### Language Mindset

According to Robinson (2017), mindset is defined as an emotional factor that signifies attitudes about their flexibility of astuteness, talents, or capabilities to perform skills. Yeager and Dweck (2012) also mentioned that mindsets are considered the fundamental suppositions individuals make about their characteristics. Regarding the educational contexts, Mercer (2012) states that foreign language mindset “reflects the extent to which a person believes that language learning ability is dependent on some immutable, innate talent or is the result of controllable factors such as effort and conscious hard work.” (p. 22). Dweck (2008) highlighted the importance of learners’ mindsets, and she claimed that mindsets form the consecutive accounts that happen in learners’ minds. She maintained that mindsets influence learners’ understanding. She maintained that individuals’ mindsets differ according to their theories about the flexibility or stability of their traits. In other words, one can interpret that his trait is dynamic or that it is stable, which she denotes this category as growth mindset and fixed mindset, correspondingly. Lou and Noels (2016) argued that learners with growth mindset regard their capability as flexible which can develop through diligence and operational approaches, whereas learners with fixed mindset think that their learning capacity is inborn, and it cannot be developed through experiencing in educational contexts. Moreover, they also argued that learners’ language learning mindsets regulate their assumptions about general language intelligence, age sensitivity in language learning and foreign language aptitude. They mentioned that the beliefs about general language intelligence and foreign language aptitude encompass learners’ assumptions about their native and foreign language capabilities, namely, whether their native and foreign language capabilities are static or dynamic. They maintained that sensitivity in language learning focuses on the critical period theory among students. The new categorizations of mindset afford a new viewpoint for examining learner’ opinions about language learning and, therefore, has drawn the attention of investigators in educational contexts (Sadeghi et al., 2020; Janudom, 2021; Yao et al., 2021). Although the prevalent opinions about the normal, inborn aptitude for language learning have been widely discussed (Mercer and Ryan, 2010), there are moderately few studies on language learning mindsets in applied linguistics. Lou and Noels (2020) found out that language mindset is significantly correlated with EFL learners’ academic achievements. Papi et al. (2019) also found out that learners’ communication apprehension is significantly correlated with their language learning mindsets. Haimovitz and Dweck (2017) argued that learners’ mindsets are affected by their rapport with instructors. Ciaccio (2019) found out that learners’ growth mindsets significantly predict their self-efficacy. He argued that students with a growth mindset believe that their negative functions in language tasks are due to their lack of determination. He also argued that learners with growth mindset try hard to do their responsibilities efficaciously. Mrazek et al. (2018), in their study, revealed that learners with a growth mindset are inclined to self-regulate their thoughts and affections. In addition, they found out that learners’ understandings of effort influence their self-regulation. Zhao et al. (2021) study revealed that learner academic engagement is significantly correlated with learner mindset. They also mentioned that stress mediates the correlation between learning engagement and growth mindset. Marlow (2021), in his study, argued that learners can decrease foreign language anxiety by transforming their fixed mindsets into growth mindsets. They argued that learners with stable mindsets have higher levels of foreign language anxiety and lower levels of performance in language learning. Zarrinabadi et al. (2021a) also found out that learners’ language mindset is significantly correlated with their communicative competence and their willingness to communicate. Zarrinabadi et al. (2021b) revealed that learners with growth mindsets are more likely to choose effective learning strategies to help foster their capability. Lou et al. (2017) also argued that language learners’ mindsets are significantly correlated with their internal motivations for language learning. They argued that language learners with learning objectives are inclined to be motivated, as they like the learning process.

### The Concept of Academic Motivation

Motivation is a widely studied issue in foreign language learning investigations. It guides various learning behaviors in numerous educational environments (Boo et al., 2015). Csizér and Dörnyei (2005) pointed out that “motivation is a concept that explains why people behave as they do rather than how successful their behavior will be” (p. 20). Dörnyei and Ushioda (2011) regard motivation as a component of enjoyment, and they mentioned that motivation “moves a person to make certain choices, to engage in action, to expend effort and persist in action” (p. 3). However, Dörnyei (2001) stated that there is an indirect relationship between academic motivation and language learning achievements since other variables such as linguistic skills, and linguistic backgrounds affect learners’ proficiency in foreign language contexts. However, he mentioned that some external negative factors like school and classroom contexts have demotivated them. Dörnyei (2005) argued that foreign language motivation indicates a multifaceted psychological notion that drives a learner to learn a second or foreign language. Alrabai and Moskovsky (2016) showed that motivation, autonomy, viewpoint, anxiety, and self-esteem are critical in L2 learning proficiency. They stated that academic motivation has the highest predictability power over other factors in language learning proficiency. Crookes and Schmidt (1991) highlighted the importance of regarding four motivational components of Keller (1983) educational orientation theory, including (a) interest, (b) relevance, (c) expectancy, and (d) satisfaction. They maintained that these components of motivation should be taken into account by instructors when they are trying the appreciate learners’ learning motivation since these components may affect learners’ language learning (Crookes and Schmidt, 1991).

The integrative and instrumental motivations have been specified as two leading components of L2 learning motivation (Fan and Feng, 2012; Carrió-Pastor and Mestre, 2014; Quan, 2014). Motivation is a blend of internal and external forces that urges learners to focus on the learning process (Gardner, 2010). Learners’ integrative motivation refers to their willingness to integrate themselves into a spoken language society. Moreover, learners’ positive viewpoints toward the teaching-learning context determine integrative and academic achievement (Khodadad and Kaur, 2016). Pavelescu (2019) found out that learners’ integrative motivation is affected by teachers’ motivation, encouragement, and support. Moreover, Cocca and Cocca (2019) found out that integrative motivation is positively correlated with language learning achievement by encouraging learners to use language effectively, and it is also related to learners’ culture. Liu (2021) stated that second language learning is significantly affected by integrative rather than instrumental correlation. He argued that motivated learners attempt to find chances to develop their language skills.

Instead, Gardner and Lambert (1972) (p. 150) pointed out that “instrumental orientation is considered a desire to gain social recognition or economic advantages through knowledge of a foreign language.” Cheng et al. (2014) asserted that higher instrumental motivation lowers test scores. Lukmani (1972) study showed that Indian EFL learners’ instrumental motivation can significantly correlate with language proficiency scores. Sallang and Ling (2019) found a relationship between providing constructive feedback to students and instrumental motivation in language learning as it develops learners’ motivation in performing tasks. Safotso and Tompte (2018) study revealed that learners with high levels of instrumental motivation are more interested in communicative approach in language learning.

Overall, Gardner (2001) asserted that some factors such as positive attitudes toward learning context, instrumental, and integrative-oriented motivations significantly affect learners’ attention, and the desire to regulate learners’ endeavors in language learning. Khalid (2016) found out that instrumental and integrative motivation in language learning is associated with learners’ socioeconomic position. He also asserted that attitudes toward language learning can be affected by these orientations. Sediqifar and Khaleghizadeh (2016) study revealed that learners’ integrative motivation is positively correlated with their academic achievement. Nevertheless, their study did not improve the significant correlation between learners’ instrumental motivation and language learning achievements.

### The Role of English as a Foreign Language Learners’ Language Mindset in Their Grit

The relationship between EFL learners’ language mindset and grit has been studied in a few investigations. Yeager and Dweck (2012), in a study, revealed that language mindset and grit are correlated with each other, and they have a significant effect on learners’ success in educational contexts. They argued that learners’ growth mindsets develop their persistency and resilience facing educational impediments. Murphy and Dweck (2015) stated that a growth mindset can contribute learners to form their perceptions of effort, and to display their well-being when one confronts disruptions and problems. Moreover, they argued that persistence and flexibility engendered by a growth mindset are the components of academic achievement. Lee and Jang (2018) found a positive significant correlation between grit, hope, growth mindset, and self-directed learning. They also found out that hope can act as a mediator in the correlation between growth mindset and grit. Wang et al. (2018), in a study, stated that grit and growth mindset are significantly correlated with each other. They argued that these two constructs are linked to cognitive-behavioral control regions. They mentioned that grit is related to the link between areas associated with upcoming rewards, while growth mindset is related to areas like error monitoring. Bahník and Vranka (2017) found no significant correlations between growth mindset, grit, and academic achievement. Bazelais et al. (2018) also mentioned that mindset and grit are not significantly correlated with academic achievement. They argued that learners’ grand point average is extensively determined as a significant component in academic achievement, and learners’ grit and mindsets overlap with their grand point average. Sigmundsson et al. (2020) found a gender effect on the relationship between mindset growth and grit. Kannangara et al. (2018) also mentioned that learners with higher levels of grittiness had significantly higher levels of resilience and self-control, and they tend to have a growth mindset. Moreover, Kench et al. (2016) examined the correlation between grit and mindset among children and adolescents, and they found out that their growth mindsets are significantly correlated with their grittiness. Duckworth (2016) also argued that learners with a growth mindset have persistence and commitments to reach their objectives. They argued that growth mindset is considered a helpful construct that advances learners’ grittiness. Khajavy et al. (2020) found out that a growth mindset is significantly correlated with perseverance as an element of grit. Based on neuroscience, Myers et al.’s (2016) study also revealed that a growth mindset mediates the correlation between grit and brain structures.

### The Role of English as a Foreign Language Learners’ Academic Motivation in Their Grit

Cross (2014) described grit as an ability to tolerate difficulties though preserving the wish for long-term purposes. Duckworth et al. (2007) also pointed out that grit includes “working strenuously toward challenges, maintaining effort and interest over years despite failure, adversity, and plateaus in progress” (p. 1088). They categorized this higher-order concept into two components: steadiness in interests and persistence in effort despite difficulties. Karlen et al. (2019) also highlighted these components in Grit Scale to measure grit construct.

Motivation, contrary to grit as a barely transformed idea, has been considered as a dynamic concept in educational contexts (Waninge et al., 2014). Some studies have been done on the relationship between grit and motivation. Ryan and Deci (2000), in a study, revealed that learners with higher levels of intrinsic motivation have higher levels of perseverance in their academic performance, and they argued that grit reflects the amount of learners’ energy in the promotion of one’s academic growth with improving their intrinsic motivation. Duckworth et al. (2007) also stated that grit, perseverance, and achievement are closely associated with each other, and learners with higher levels of grittiness tend to be motivated, determined, and successful in the educational contexts. Gyamfi and Lai (2020) argued that grit has a significant relationship with motivation along with resilience, hardness, and self-efficacy. Their study implicated that teachers can offer some approaches to foster learners’ grit and perseverance, and improve their motivation and interest in EFL classrooms to achieve new goals. Yang (2021) found out that enjoyment, as an umbrella term for motivation, is significantly correlated with learners’ grit and well-being. They argued that gritty learners perform enthusiastically at complex tasks and through this, they enjoy their academic achievements. They also justified their study using Macintyre and Gregersen (2012) broad-and-build theory. According to this theory, positive contexts with higher levels of motivation in educational classes make learners demonstrate more grittiness. Changlek and Palanukulwong (2015) revealed that also grit is negatively correlated with communication apprehension, and positively correlated with internal motivation. Steinmayr et al. (2018) study used expectancy-value theory to investigate the relationship between grit and motivational elements, and they found that learners’ grit significantly predicts their motivation, engagement, and personality. Karlen et al. (2019), in their study, pinpointed the significant role of learners’ perseverance and consistency of interest in intrinsic and extrinsic motivation. They argued that grittier learners try to find the importance and objective of a task, and to enhance their intrinsic motivation. Katherine et al. (2014) argued that learners’ grit significantly predicts their individual differences as learners’ persistence stems from their motivations. Werner et al. (2018) found a significant correlation between grit and self-regulatory traits, including persistency and consistency among EFL learners. They maintained that internal motivation accelerates learners’ persistence in performing tasks. They argued that the motivational awareness that grit produces is significant for improvement in academic performance. Bozgün and Akın-Kösterelioğlu (2021) also found out that academic grit and well-being mediate the relationship between social-emotional growth and motivation in language skills. Giordano (2019) found a correlation between grit and intrinsic motivation. He found out that the grit and intrinsic motivation are one-dimensional constructs. Duckworth and Eskreis-Winkler (2013) found out that grit is negatively correlated with anxiety, and positively correlated with willingness to communicate and motivation. Teimouri et al. (2020) studied the learners’ grit and numerous motivational, and language-related constructs. They found out that learners’ enjoyment, attention, willingness to communicate, and linguistic proficiency are significantly affected by their grit. Their study also showed that learners’ communication apprehension and grit are negatively correlated with each other.

## Concluding Remarks

This review inspected the related literature on the effect of learners’ academic motivation and growth mindsets on their grit. This review concluded that there are positive correlations between grit, mindset and motivations. It can be argued that learners can be mindful in adjusting, modifying, and controlling their emotional states in language learning contexts through attending to the related literature on the correlations among motivation and grit as affective factors. Not only can Learners improve their achievements in their academic life, but also increase their grit through increasing their integrative motivation and growth mindset. Those learners who cannot control their grit make instructors try to integrate academic motivation into the EFL context through consulting with learners and providing pleasant learning contexts. They can also improve their grit by encouraging learners to engage in classrooms and to be motivated in the EFL contexts. L2 teachers should try to find materials to improve learners’ positive attitudes and motivation for improving their grit and persistence in the effort. Teachers are required to alleviate learners’ anxiety, disengagement, and tension and to improve their grittiness and growth mindset irrespective of educational problems in language learning environments. This review implicates that teachers are needed to provide creative, interesting, and pleasant language tasks since motivating tasks can heighten learners’ cognitive resources, grit, and strengthen language learning in their minds. Therefore, the provision of pleasant and motivating activities can help learners effectively regulate their grit and learning performance.

Furthermore, EFL teachers’ knowledge about learners’ mindsets and internal motivations may stimulate teachers to help them engage in language contexts. Therefore, instructors should consider individual differences and their effect on academic performance. In other words, grit, motivation, and mindsets should be highlighted in school curricula and teacher education. Teachers can improve learners’ motivation by providing regulatory strategies to help them encounter possible disturbances, postponement, and other motivational challenges. Teacher educators and mentors can rationally take into account the conditions in language learning environments and offer some techniques for teachers to improve learners’ mindset, grittiness, and academic motivation. They can hold workshops and provide teacher courses to talk about the significance of grit in language learning. Correspondingly, this review implicates that teacher educators should be cautious about the critical role of learners’ motivation and mindset in language learning contexts. It is recommended that teacher educators be sociable, humorous, and obliged to provide new stimulating language tasks that connect learners’ language proficiency with their grit (Dewaele et al., 2019). Hence, attending to learners’ motivation, language mindset, and grit may boost students’ positive emotions may improve the collaboration among peers, increase teacher’s rapport with learners and eventually stimulate learners’ sense of enjoyment. Policymakers can also hold academic workshops which help teachers be attentive in intensifying learners’ grittiness and their academic motivation. They can create a positive learning context in which students can participate in positive behaviors. The importance of academic motivation and language mindsets make consultants extend their views and programs for developing the effect of these variables on learners’ grit. They can identify learners’ barriers in being persistent in effort. Material developers can also incorporate podcasts and videotapes into learning contexts to arouse learners’ grit and motivation. Learners’ grit and motivation can be affected by their home experiences. Therefore, parents can help them build their persistence in effort and improve their mindsets by providing them with responsibility in their life.

Some suggestions have been provided in this review. Future research can validate numerous measures of learners’ grittiness and motivation. In order to ponder into the influence of learners’ academic motivation and language mindset on their grit, a longitudinal study can also be done instead of cross-sectional studies. Besides, studies should be done on learners’ academic motivation, language mindset, and grit in numerous educational, local, national, and cultural contexts. Some investigations need to be done on the relationship between learners’ mindset, psychological well-being, and academic engagement. Future studies should be conducted on the effect of fixed and growth mindsets on learners’ academic engagement and communication apprehension. Other investigations can also be done on the relationship between fixed and growth mindsets and learners’ emotional intelligence in foreign language learning.

Regarding academic motivation, the relationship between EFL learners’ internal and external motivation and their emotional intelligence in EFL contexts can be investigated in the future. Further studies should be done on the effect of instructors’ practices on learners’ motivation. Besides, future studies should concentrate on the effect of gender on language learners’ internal and external motivation. Investigators should study the influence of video games on learners’ motivation. In addition, future investigations can examine the impacts of learner motivation on working memory. Moreover, the effects of EFL learners’ motivation on the development of language skills are required to be reflected at greater length. Additionally, future studies should examine the mutuality of the correlation between EFL learners’ motivation and grit in the future. The effect of learners’ motivation on teachers’ well-being and work engagement can also be studied. Future studies should cogitate learners’ internal motivation in traditional and virtual contexts to clarify the effect of learning contexts on learner motivation.

Concerning grit, some investigations are needed to elucidate the relationship among foreign language learners’ grit, hope, optimism, and emotional intelligence. Also, the relationship between learners’ grit and teacher burnout needs to be examined. Moreover, the relationship between learner grit and boredom is worth to be studied. Resilience, as another component of positive psychology, and its relationship with learner grit should be elaborated in greater detail. Future studies may include the relationship between learner grit and five big personality traits.

## Ethics Statement

The studies involving human participants were reviewed and approved by Henan Institute of Technology Academic Ethics Committee. The patients/participants provided their written informed consent to participate in this study.

## Author Contributions

The author confirms being the sole contributor of this work and has approved it for publication.

## Conflict of Interest

The author declares that the research was conducted in the absence of any commercial or financial relationships that could be construed as a potential conflict of interest.

## Publisher’s Note

All claims expressed in this article are solely those of the authors and do not necessarily represent those of their affiliated organizations, or those of the publisher, the editors and the reviewers. Any product that may be evaluated in this article, or claim that may be made by its manufacturer, is not guaranteed or endorsed by the publisher.

## References

[B1] Alrabai B. Moskovsky C. (2016). The relationship between learners’ affective variables and second language achievement. *Arab World English J. (AWEJ)* 7 11–21. 2 10.2139/ssrn.2814796

[B2] Arabmofrad A. Derakhshan A. Atefinejad M. (2019). An interplay between Iranian EFL learners’ specific and general interlanguage pragmatic motivation and their meta-pragmatic awareness. *Iran. J. Engl. Acad. Purp.* 8 77–94.

[B3] Bahník Š Vranka M. (2017). Growth mindset is not associated with scholastic aptitude in a large sample of university applicants. *Pers. Individ. Differ.* 117 139–143. 10.1016/j.paid.2017.05.046

[B4] Boo Z. Dörnyei Z. Ryan S. (2015). L2 motivation research 2005–2014: understanding a publication surge and a changing landscape. *System* 55 145–157. 10.1016/j.system.2015.10.006

[B5] Bozgün and Akın-Kösterelioğlu M. (2021). The roles of social-emotional development, academic grit and subjective well-being in primary school students’ reading-writing motivation. *Read. Psychol.* 42 836–857. 10.1080/02702711.2021.1955782

[B6] Bazelais P. Lemay D. J. Doleck T. Hu X. S. Vu A. Yao J. (2018). Grit, mindset, and academic performance: a study of pre-university science students. *Eurasia J. Math. Sci. Technol. Educ.* 14 1–10. 10.29333/ejmste/94570

[B7] Carrió-Pastor M. L. Mestre E. M. M. (2014). Motivation in second language acquisition. *Procedia-Soc. Behav. Sci.* 116 240–244.

[B8] Changlek A. Palanukulwong T. (2015). Motivation and grit: predictors of language learning achievement. *Veridian E-Journal.* 8 23–38.

[B9] Cheng L. Klinger D. Fox J. Doe C. Jin Y. Wu J. (2014). Motivation and test anxiety in test performance across three testing contexts: The CAEL. CET and GEPT. *TESOL Quarterly* 48 300–330. 10.1002/tesq.105

[B10] Ciaccio B. (2019). *Should we give a Grit About Movement? Examining the Relationships Among Mindset, Grit, Self-Efficacy, and Exercise Behavior*, Doctoral dissertation. Philadelphia, PA: Temple University.

[B11] Cocca M. Cocca A. (2019). Affective variables and motivation as predictors of proficiency in English as a foreign language. *J. Effici. Res. Educ. Sci.* 12 75–83. 10.7160/eriesj.2019.120302 29090908

[B12] Crookes G. Schmidt R. W. (1991). Motivation: reopening the research agenda. *Lang. Learn.* 41 469–512. 10.1111/j.1467-1770.1991.tb00690.x

[B13] Cross T. (2014). The gritty: grit and non-traditional doctoral student success. *J. Educ. Online* 11 3–24. 10.9743/JEO.2014.3.4

[B14] Csizér K. Dörnyei Z. (2005). The internal structure of language learning motivation and its relationship with language choice and learning effort. *Modern Langu. J.* 89 19–36. 10.1111/j.0026-7902.2005.00263.x

[B15] Dewaele J.-M. Chen X. Padilla A. M. Lake J. (2019). The flowering of positive psychology in foreign language teaching and acquisition research. *Front. Psychol.* 10:2128. 10.3389/fpsyg.2019.02128 31607981 PMC6769100

[B16] Dörnyei Z. (2001). New themes and approaches in second language motivation research. *Annual. Rev. Appl. Linguist.* 21 43–59. 10.1017/S0267190501000034

[B17] Dörnyei Z. (2005). *The Psychology Of the Language Learner. Individual Differences In Second Language Acquisition.* Mahwah: Lawrence Erlbaum.

[B18] Dörnyei Z. Ushioda E. (2011). *Teaching and Researching Motivation.* London:UK: Pearson.

[B19] Duckworth A. L. Peterson C. Matthews M. D. Kelly D. R. (2007). Grit: Perseverance and passion for long-term goals. *J. Pers. Soci. Psychol.* 92 1087–1101. 10.1037/0022-3514.92.6.1087 17547490

[B20] Duckworth A. L. Eskreis-Winkler L. (2013). True grit. *Observer* 26. Available online at: https://www.psychologicalscience.org/observer/true-grit (accessed April 3, 2022).

[B21] Duckworth A. L. (2016). *Grit: The Power of Passion and Perseverance.* New York, NY: Scribner.

[B22] Dweck C. S. (2008). *Mindsets and Math/Science Achievement.* New York, NY: Carnegie Corporation of New York.

[B23] Elizondo L. B. (2013). The mixed-proficiency language class: consequences for students. *Letras* 53 111–135. 10.15359/rl.1-53.5

[B24] Fan J. J. Feng H. Y. (2012). A study on students’ learning motivation of EFL in Taiwanese vocational college. *Int. J. Learn. Dev.* 2 260–269. 10.5296/ild.v2i3.1791

[B25] Fosnacht K. Copridge K. Sarraf S. A. (2019). How valid is grit in the postsecondary context? a construct and concurrent validity analysis. *Res. Higher Educ.* 60 803–822. 10.1007/s11162-018-9524-0

[B26] Gardner R. C. Lambert W. E. (1972). *Attitudes and Motivation In Second Language Learning.* Rowley, MA: Newbury House.

[B27] Gardner R. C. (2001). “Integrative motivation and second language acquisition,” in *Motivation and Second Language Learning*, eds Dörnyei Z. Schmidt R. (Honolulu, HI: University of Hawai Press), 1–20. 10.1111/j.1467-1770.1984.tb00339.x

[B28] Gardner R. C. (2010). *Motivation and Second Language Acquisition: The Socio-Educational Model.* New York, NY: Peter Lang.

[B29] Giordano M. J. (2019). Grit and intrinsic motivation for language learning: instrument validation using the rasch model. *Shiken* 23 38–42.

[B30] Gyamfi G. Lai Y. (2020). Beyond motivation: investigating thai english major students’ grit. *PASAA* 60 60–96.

[B31] Haimovitz K. Dweck C. S. (2017). The origins of children’s growth and fixed mindsets: new research and a new proposal. *Child Dev.* 88 1849–1859. 10.1111/cdev.12955 28905371

[B32] Janudom R. (2021). Raising teachers’ awareness of students’ mindsets in EFL learning. *New Eng. Teac.* 15 1–8. 10.1080/09588221.2019.1629962

[B33] Kannangara C. S. Allen R. E. Waugh G. Nahar N. Khan S. Rogerson S. (2018). All that glitters is not grit: Three studies of grit in university students. *Front. psychol.* 9:1–15. 10.3389/fpsyg.2018.01539 30210389 PMC6123604

[B34] Karabatak S. Polat H. (2020). The effects of the flipped classroom model designed according to the ARCS motivation strategies on the students’ motivation and academic achievement levels. *Educ. Inf. Technol.* 25 1475–1495. 10.1007/s10639-019-09985-1

[B35] Karlen Y. Suter F. Hirt C. Merki K. M. (2019). The role of implicit theories in students’ grit and mindset in language learning grit, achievement goals, intrinsic and extrinsic motivation, and achievement in the context of a long-term challenging task. *Learn. Individ. Differ.* 74 101–757. 10.1016/j.lindif.2019.101757

[B36] Katherine V. C. Tsukayama E. Duckworth A. (2014). Unpacking grit: motivational correlates of perseverance and passion for long-term goals. *J. Posit. Psychol.* 9 1–9. 10.1080/17439760.2014.898320 31404261 PMC6688745

[B37] Keller J. M. (1983). “Motivational design of instruction,” in *Instructional design theories and models*, ed. Riegeluth C. M. (Hillsdale, NJ: Lawrence Erlbaum), 383–434.

[B38] Kench D. Hazelhurst S. Otulaja F. (2016). “Grit and growth mindset among high school students in a computer programming project: A mixed methods study,” in *Proceedings of Annual Conference of the Southern African Computer Lecturers’ Association*, ed. Gruner S. (New York, NY: Springer), 187–194. 10.1007/978-3-319-47680-3_18

[B39] Khajavy G. H. Macintyre P. Hariri J. (2020). A closer look at grit and language mindset as predictors of foreign language achievement. *Stud. Second Lang. Acquis.* 5 1–54. 10.1017/S0272263120000480

[B40] Khalid A. (2016). *A study of the Attitudes and Motivational Orientations Of Pakistani Learners Toward The Learning Of English as a Second Language.* 6. Thousand Oaks: SAGE, 10.1017/2158244016665887

[B41] Khodadad M. Kaur J. (2016). Causal relationships between integrative motivation, self- efficacy, strategy use and English language achievement. 3L-language linguistics literature. *Southeast Asian J. Eng. Lang. Stud.* 22 111–125. 10.17576/3L-2016-2203-08

[B42] Lee C. S. Jang H. Y. (2018). The roles of growth mindset and grit in relation to hope and self-directed learning. *J. Korea Conv. Society* 9 95–102. 10.15207/JKCS.2018.9.1.095

[B43] Liu W. (2021). Does teacher immediacy affect students? A systematic review of the association between teacher verbal and non-verbal immediacy and student motivation. *Front. psychol.* 12 1–13. 10.3389/fpsyg.2021.713978 34248809 PMC8267458

[B44] Lou N. M. Masuda T. Li L. M. W. (2017). Decremental mindsets and prevention-focused motivation: an extended framework of implicit theories of intelligence. *Learn. Individ. Differ.* 59 96–106. 10.1016/j.lindif.2017.08.007

[B45] Lou N. Noels K. (2016). Changing language mindsets: implications for goal orientations and responses to failure in and outside the second language classroom. *Contemp. Educ. Psychol.* 46 22–33. 10.1016/j.cedpsych.2016.03.004

[B46] Lou N. Noels K. (2020). Breaking the vicious cycle of language anxiety: growth language mindsets improve lower-competence ESL students’ intercultural interactions. *Contemp. Educ. Psychol.* 61 1–23. 10.1016/j.cedpsych.2020.101847

[B47] Lukmani M. Y. (1972). Motivation to learn and language proficiency. *Lang. Learn.* 22 261–273. 10.1111/j.1467-1770.1972.tb00087.x

[B48] Macintyre P. Gregersen T. (2012). Emotions that facilitate language learning: the positive-broadening power of the imagination. *Stud. Second Lang. Learn. Teach.* 2 193–213. 10.14746/ssllt.2012.2.2.4

[B49] Mammadov S. Cross T. L. Olszewski-Kubilius P. (2021). A look beyond aptitude: the relationship between personality traits, autonomous motivation, and academic achievement in gifted students. *Roeper Rev.* 43 161–172. 10.1080/02783193.2021.1923595

[B50] Marlow B. (2021). *Second Language Learning Anxiety and Language Mindsets. Senior Honors Theses.* Virginia: US: Liberty University.

[B51] Mercer S. (2012). Dispelling the myth of the natural-born linguist. *ELT J.* 62 22–29. 10.1093/elt/ccr022

[B52] Mercer S. Ryan S. (2010). A mindset for EFL: Learners’ beliefs about the role of natural talent. *ELT J.* 64 436–444. 10.1007/s10936-021-09787-y 34269960

[B53] Mrazek A. J. Ihm E. D. Molden D. C. Mrazek M. D. Zedelius C. M. Schooler J. W. (2018). Expanding minds: growth mindsets of self-regulation and the influences on effort and perseverance. *J. Exp. Soc. Psychol.* 79 164–180. 10.1016/j.jesp.2018.07.003

[B54] Murphy M. Dweck C. (2015). Mindsets shape consumer behavior. *J. Consum. Psychol.* 26 127–136. 10.1016/j.jcps.2015.06.005

[B55] Myers C. A. Wang C. Black J. M. Bugescu N. Hoeft F. (2016). The matter of motivation: striatal resting-state connectivity is dissociable between grit and growth mindset. *Soc. Cogn. Affect. Neurosci.* 11, 1521–1527. 10.1093/scan/nsw065 27217105 PMC5040906

[B56] Papi M. Rios A. Pelt H. Ozdemir E. (2019). Feedback-seeking behavior in language learning: basic components and motivational antecedents. *Mod. Lang. J.* 103 205–226. 10.1111/modl.12538

[B57] Pavelescu L. M. (2019). Motivation and emotion in the EFL learning experience of Romanian adolescent students: two contrasting cases. *Stud. Second Lang. Learn. Teach.* 9 55–82. 10.14746/ssllt.2019.9.1.4

[B58] Plaks J. E. Stecher K. (2007). Unexpected improvement, decline, and stasis: a prediction confidence perspective on achievement success and failure. *J. pers. soc. psychol.* 93:667. 10.1037/0022-3514.93.4.667 17892338

[B59] Quan Z. (2014). Motivation for a second or foreign language learning. *SHS Web Conf.* 6:A04004. 10.1051/shsconf/20140604004

[B60] Robinson C. (2017). Growth mindset in the classroom. *Sci. Scope* 41 18–21.

[B61] Ryan R. M. Deci E. L. (2000). Self-determination theory and the facilitation of intrinsic motivation, social development, and well-being. *Am. Psychol. J.* 55 68–78. 10.1037/0003-066x.55.1.68 11392867

[B62] Sadeghi F. Sadighi F. Bagheri M. S. (2020). The relationship between Iranian EFL learners’ language mindset with goal orientation and responses to failure. *Cogent Educ.* 7 1–22. 10.1080/2331186X.2020.1833814

[B63] Safotso G. T. Tompte N. (2018). Attitudes and motivation of chadian learners of english. *World J. Educ.* 8 174–180. 10.5430/wje.v8n2p174

[B64] Sallang H. Ling Y. L. (2019). The importance of immediate constructive feedback on students’ instrumental motivation in speaking in english. *Britain Int. Lin. Arts Educ. (BIoLAE) J.* 1 1–7. 10.33258/biolae.v1i2.58

[B65] Saunders S. A. (2013). *The Impact Of A Growth Mindset Intervention On The Reading Achievement Of At-Risk Adolescent Students.* Ann Arbor, MI: ProQuest LLC.

[B66] Sediqifar Z. Khaleghizadeh S. (2016). Motivational orientations and academic achievement among Arabic speaking learners of persian. *J. Teach. Pers. Lang. Non-Pers. Speak.* 5 75–93.

[B67] Sharkey C. M. Bakula D. M. Baraldi A. N. Perez M. N. Suorsa K. I. Chaney J. M. (2018). Grit, illness-related distress, and psychosocial outcomes in college students with a chronic medical condition: a path analysis. *J. pediatr. psychol.* 43 552–560. 10.1093/jpepsy/jsx145 29240936

[B68] Sigmundsson H. Haga M. Hermundsdottir F. (2020). Passion, grit and mindset in young adults: exploring the relationship and gender differences. *New Ideas Psychol.* 59 1–7. 10.1016/j.newideapsych.2020.100795

[B69] Steinmayr R. Weidinger A. F. Wigfield A. (2018). Does students’ grit predict their school achievement above and beyond their personality, motivation, and engagement? *Contemp. Educ. Psychol.* 53 106–122. 10.1016/j.cedpsych.2018.02.004

[B70] Teimouri Y. Plonsky L. Tabandeh F. (2020). L2 grit: Passion and perseverance for second-language learning. *Lang. Teach. Res.* 107 1–18. 10.1177/1362168820921895

[B71] Tough P. (2012). *How Children Succeed: Grit, curiosity, and the Hidden Power Of Character.* Boston: Houghton Mifflin Harcourt.

[B72] Vela J. C. Smith W. D. Whittenberg J. F. Guardiola R. Savage M. (2018). Positive psychology factors as predictors of latina/o college students’ psychological grit. *J. Multicult. Couns. Devel.* 46 2–19. 10.1002/jmcd.12089

[B73] Waninge F. Dörnyei Z. De Bot K. (2014). Motivational dynamics in language learning: change, stability, and context. *Mod. Lang. J.* 98 704–723.

[B74] Weiner B. (1985). An attributional theory of achievement motivation and emotion. *Psychol. Rev.* 92 548–573. 10.1037/0033-295x.92.4.548 3903815

[B75] Wang S. Dai J. Li J. Wang X. Chen T. Yang X. (2018). Neuroanatomical correlates of grit: growth mindset mediates the association between gray matter structure and trait grit in late adolescence. *Hum. Brain Mapp.* 39 1688–1699. 10.1002/hbm.23944 29331059 PMC6866491

[B76] Werner K. M. Milyavskaya M. Klimo R. Levine S. L. (2018). Examining the unique and combined effects of grit, trait self-control, and conscientiousness in predicting motivation for academic goals: a commonality analysis. *J. Res. Pers.* 81 168–175. 10.1016/j.jrp.2019.06.003

[B77] Yang P. (2021). Exploring the relationship between Chinese EFL students’ grit, well-being, and classroom enjoyment. *Front. psychol.* 12 1–19. 10.3389/fpsyg.2021.762945 34777167 PMC8586070

[B78] Yao Y. Guo N. S. Wang W. Yu J. (2021). Measuring Chinese junior high school students’ language mindsets: what can we learn from young EFL learners’ beliefs in their language ability? *System* 101 1–15. 10.1016/j.system.2021.102577

[B79] Yeager D. S. Dweck C. S. (2012). Mindsets that promote resilience: when students believe that personal characteristics can be developed. *Educ. Psychol.* 47 302–314. 10.1080/00461520.2012.722805

[B80] Zarrinabadi N. Lou N. M. Shirzad M. (2021a). Autonomy support predicts language mindsets: implications for developing communicative competence and willingness to communicate in EFL classrooms. *Learn. Individ. Differ.* 86 1–8. 10.1016/j.lindif.2021.101981

[B81] Zarrinabadi N. Rezazadeh M. Chehrazi A. (2021b). The links between grammar learning strategies and language mindsets among L2 and L3 learners: Examining the role of gender. *Int. J. Multiling.* 1–18. 10.1080/14790718.2020.1871356.

[B82] Zhao H. Xiong J. Zhang Z. Qi C. (2021). Growth mindset and college students’ learning engagement during the COVID-19 pandemic: a serial mediation model. *Front. Psychol.* 12 1–10. 10.3389/fpsyg.2021.621094 33679536 PMC7933026

